# Synthesis and Cytotoxicity Study of New Cyclopenta [b] quinoline-1,8-dione Derivatives

**Published:** 2011

**Authors:** Ramin Miri, Omidreza Firuzi, Payam Peymani, Zohreh Nazarian, Abbas Shafiee

**Affiliations:** a *Medicinal and Natural Products Chemistry Research Center, Shiraz University of Medical Sciences. Shiraz, Iran*; b *Pharmaceutical Sciences Research Center, Tehran University of Medical Sciences. Tehran, 14174, Iran*; c *Department of Medicinal Chemistry, Faculty of Pharmacy, Tehran University of Medical Sciences*, *Tehran, Iran. *

**Keywords:** Cytotoxicity, Cyclopenta [b] quinoline-1, 8-dione, MTT assay, DNA

## Abstract

DNA intercalators belong to aromatic heterocyclic compounds interacting reversibly with DNA. These compounds have been used extremely as cytotoxic agents against cancer. In this study, the synthesis and biological activity of some novel derivatives of cyclopenta [*b*] quinoline-1, 8-dione as new intercalating agent were investigated. Twenty novel derivatives of cyclopenta [*b*] quinoline-1, 8-dione were synthesized by molecular condensation of equivalent amount of 3-imino cyclopentanone, corresponding aldehyde and cyclohexane-1, 3-dione. Then, their cytotoxic activity was evaluated against HeLa, LS180, MCF-7 and Raji cancer cell lines by MTT assay.

The results of cytotoxic activity evaluation indicate that the most of synthesized compounds show weak cytotoxic effect on the different cell lines (IC_50_ of these compounds is higher than 50 or 100 µ ). According to previous studies, in the case of compounds with the weak biological activity, it is more suitable to use IC_15 _and IC_30_ instead of IC_50_ as the indicator of biological activity. Since most of compounds have weak cytotoxic effect, we also calculated IC_15_ and IC_30_ for evaluating the cytotoxic activity of synthesized compounds. The most potent compound, 6 h (9-(3-Bromo-phenyl)-4-pheny l-2, 3, 5, 6, 7, 9-hexahydro-4H-cyclopenta [*b*] quinoline-1, 8-dione), containing bromophenyl moiety and phenyl substitute on nitrogen of central quinoline ring, show significant cytotoxic activity especially in Raji and HeLa cell lines (IC_30_: 82 and 24.4 μ M respectively) comparing to other compounds. Although the results of cytotoxic activity evaluation demonstrated that the *in-vitro *anti-cancer effect of synthesized compounds are mainly low, it seems that this structure can be used as a novel cytotoxic scaffold for further modification and design of novel potent compounds.

## Introduction

Fifty years ago, Watson and Crick discovered that DNA is structurally present as a double helix (1). Since this genetic molecule has power over the cellular functions, it is mentioned as an excellent target for treating genetic-based disorders, like cancer. In the 1960s, some compounds with anti-cancer capacity were synthesized to act as chemotherapeutic agents. Lerman *et al. *demonstrated that the cytotoxicity of those compounds is a result of non-covalent interaction between acridine and DNA, suggesting an intercalative process. Nowadays, It has been established that some of chemotherapeutic agents work by interacting with DNA (2-5). 

Generally, DNA interactions can be classified into two main classes: intercalation and groove binding (6). In intercalation process, a planar molecule can be inserted between DNA base pairs which leads to a decrease in the DNA helical twist and lengthening of the DNA (4, 7). The intercalation mechanisms start with the transfer of the intercalating agents from an aqueous media to the hydrophobic area of inter-DNA base pairs. This process leads to deformation of the sugar-phosphate structure and conversion in the angles between successive base pairs. Once, the therapeutic molecules have been sandwiched into the DNA base pairs, the stability of the DNA-molecule complex is optimized by a number of non-covalent interactions, like van der Waals and *π*-stacking bonds (8). Finally, DNA intercalation leads to suppression of the DNA replication and gene transcription, therefore, these agents can be used to destroy cancer (9).

DNA intercalators belong to aromatic heterocyclic compounds which interact reversibly with DNA (10, 6). The flat structure of these ligands intercalate between pairs of DNA molecules and share usual backbone characteristics like the presence of planar polyaromatic systems that penetrating between DNA base-pairs vertically (perpendicularly) and bond non-covalently with it (11-14). In this way, some novel polycyclic condensed systems including quinoline, pyridine and pyrimidine rings were reported as potent intercalating agents (9, 15-17). Derivatives of tetrahydropyrrolo [3, 4-a]-carbazole-1, 3-dione and tetrahydropyrido [3, 2*-b*] pyrrolo [3, 4-g] indole-1, 3-dione demonstrated significant cytotoxicity, DNA intercalation, and topoisomerase II inhibition activity (18). Furthermore, 5, 11-Dimethyl- 5H-indole [2, 3*-b*] quinoline showed a potent antimycotic, and cytotoxic efficacy (19). In addition, new class of tetracyclic 11-oxo-11-H*-*indeno [1, 2-*b*]quinoline-6-carboxamide was examined and showed good cytotoxic activity and potential dual topoisomerase I and II inhibiting activity (20). 

Therefore, in this study we proposed to synthesize novel derivatives of cyclopenta [*b*] quinoline-1, 8-dione as new intercalating agents and evaluate their cytotoxic properties in different cancer cell lines. 

## Experimental

*Chemistry*

*General procedure for synthesis of compounds*

The synthesis of tetrahydro-5-H-cyclopenta [*b*] quinoline-1, 8-dione, hexahydro-4*H*-cyclopenta [*b*] quinoline-1, 8-dione or tetrahydro-4H-cyclopenta [*b*] quinoline-1, 8 (5*H*,9*H*)-dione derivatives was achieved following the steps outlined in Scheme 1. The hexahydro analogues 4a-h were synthesized by molecular condensation of equivalent amount of 3-imino cyclopentanone 1, corresponding aldehyde 2a-h and cyclohexane-1, 3-dione 3. The hexahydro analogues 6c-h were synthesized by similar molecular condensation in which the equivalent amount of (Z)-3-(phenylimino)cyclopentanone 5, corresponding aldehyde 2c-h and cyclohexane-1, 3-dione 3 has been reacted. Then, the tetrahydro-4H-cyclopenta [*b*] quinoline-1, 8 (5H, 9H)-dione 7a-f derivatives were achieved by oxidizing the corresponding tetrahydro-5H-cyclopenta [*b*] quinoline-1, 8-dione form using MnO_2_. These compounds were purified by preparative thin layer chromatography and recrystalization, and then characterized by mass spectroscopy, IR and ^1^H NMR.

*9-(5-Bromothiophen-2-yl)-2, 3, 6, 7-tetrahydro-4H-cyclopenta [b] quinoline-1, 8 (5H,9H)-dione (4a). (C*_16_ *H*_14_ *BrNO*_2_ *S)*

1H-NMR (CDCl_3_): δ 1.95-2.54 (m, 10H, W4Hz C5-thiophene), 6.89 (d, 1H, J = 4Hz , C4-thiophene); MS: m/z (%) 363/365 (M^+^/M^+2^, 20/20), 284 (100), 225 (92), 202 (48), 199 (8), 117 (5), 56 (5); IR (KBr): ν (cm^-1^) 3416, 3021, 1736, 1629

*9-(5-Bromo-thiophen-2-yl)-2, 3, 6, 7-tetrahydro-5H-cyclopenta [b] quinoline-1, 8-dione (7a). (C*_16_ *H*_12_ *BrNO*_3_ *S)*

1H-NMR (CDCl_3_): δ 2.1-3.25(m, 10H, Aliphatic), 6.76 (d, 1H, J = 8Hz ,H_3_-furyl), 7.13(d, 1H, J = 8Hz , H_4_-furyl)

MS: m/z (%) 361/363 ((M^+^/M^+2^, 20/20), 282 (100), 254 (10), 238 (5), 171 (2)

IR (KBr): ν (cm^-1^) 3446, 2924, 1726, 1680, 1541

*9-(Furan-2-yl)-2, 3, 6, 7-tetrahydro-4H-cyclopenta [b] quinoline-1, 8 (5H,9H)-dione (4b). (C*_16_ *H*_15_ *NO*_3_ *)*

^1^H-NMR (CDCl_3_): δ 1.83-2.66 (m, 10H, Aliphatic), 4.75 (s, 1H, H-C9), 5.88 (d, 1H, , J = 3Hz , C5-furan), 6.22 (dd, 1H, C4-furan), 7.34-7.42 (m, 1H, C3-furan), 10.08 (s, 1H, H-NH); MS: m/z (%) 269 (M^+^, 79), 239 (100), 192 (20), 167 (40), 102 (20)

IR (KBr): ν (cm^-1^) 3262, 2919, 1639

*9-(Furan-2-yl)-2, 3 ,6, 7-tetrahydro-5H-cyclopenta [b] quinoline-1, 8-dione (7b). (C*_16_ *H*_13_ *NO*_3_ *)*

^1^H-NMR (CDCl_3_): δ 2.2-3.3(m, 10H, Aliphatic), 6.63 (d, 1H, J = 3.5Hz, H_3_-furyl), 7.21-7.24 (dd, 1H, H_4_-furyl), 7.56 (d, 1H, J = 3.5Hz, H_4_-furyl), MS: m/z (%) 267 (M^+^, 40), 239 (100), 210 (16), 154 (10), 128 (2); IR (KBr): ν (cm^-1^) ν 3431, 2919, 2356, 1695, 1547

*9-(3-Methoxyphenyl)-2, 3 ,6, 7-tetrahydro-4H-cyclopenta [b] quinoline-1, 8 (5H,9H)-dione (4c). (C*_19_ *H*_19_ *NO*_3_ *)*

^1^H-NMR (CDCl_3_): δ 1.95-2.63 (m, 10H, Aliphatic), 3.67 (s, 3H, H-OCH_3_), 4.63 (s, 1H, H-C9), 6.638-6.721 (m, 3H, C9-phenl), 7.071-7.107 (m, 1H, C9-phenyl), 10.02 (s, 1H, H-NH), MS: m/z (%) 309 (M^+^, 50), 305 (40), 202 (100), 201 (97); IR (KBr): ν (cm^-1^) 3441, 2924, 1639

*9-(3-Methoxy-phenyl)-4-phenyl-2, 3, 5, 6, 7, 9-hexahydro-4H-cyclopenta [b] quinoline-1, 8-dione (6c). (C*_25_ *H*_23_ *NO*_3_ *)*

^1^H-NMR (CDCl_3_): δ 1.92-2.42 (m, 10H, Aliphatic), 3.81 (s, 3H, H-methyl), 5.11 (s, 1H, H-C9), 6.69-7.54 (m, 9H, Aromatic); MS: m/z (%) 400 (M^+^, 18), 383 (78), 323 (30), 277 (100); IR (KBr): ν (cm^-1^) 3441, 2914, 1685, 1639

*9-(3-Methoxy-phenyl)-2, 3, 6, 7-tetrahydro-5H-cyclopenta [b] quinoline-1, 8-dione (7c). (C*_19_ *H*_17_ *NO*_3_ *)*

^1^H-NMR (CDCl_3_): δ 2.1-3.3 (m, 10H, Aliphatic), 3.8 (s, 3H, H-CH_3_), 6.580 (s, 1H, H2-phenyl), 6.690-6.706 (d, 1H, J = 8Hz, H4-phenyl), 6.95-7.06 (dd, 1H, H_6_-phenyl), 7.26 (s, 1H, H_2_-phenyl), 7.32 (dd, 1H, H_5_-phenyl)

MS: m/z (%) 307 (M^+^, 100), 294 (40), 210 (16), 251 (10), 219 (5); IR (KBr): ν (cm^-1^) 3416, 2919, 1721, 1690, 1536

*9-(4-Methoxyphenyl)-2, 3 ,6, 7-tetrahydro-4H-cyclopenta [b] quinoline-1, 8 (5H,9H)-dione (4d). (C*_19_ *H*_19_ *NO*_3_ *)*

^1^H-NMR (CDCl_3_): δ 2.20-2.50 (m, 10H, Aliphatic), 3.66 (s, 3H, H-OCH_3_), 4.58 (s, 1H, H-C9), 6.72 (d, 2H, J = 9Hz , C9-H_3,5_-phenyl), 7.03 (d, 2H, J = 9Hz , C9-H_2,6_-phenyl), 9.98 (S, 1H, H-NH), MS: m/z (%) 309 (M^+^, 40), 252 (17), 201 (100), 145 (15); IR (KBr): ν (cm^-1^) 3439, 2929, 1689

*9-(4-Methoxy-phenyl)-4-phenyl-2, 3, 5, 6, 7, 9-hexahydro-4H-cyclopenta [b] quinoline-1, 8-dione (6d). (C*_25_ *H*_23_ *NO*_3_ *)*

^1^H-NMR (CDCl_3_): δ 1.86-2.31 (m, 10H, Aliphatic), 3.75 (s, 3H, H-methyl), 5.05 (s, 1H, H-C9), 6.81-6.87 (m, 4H, C9-H_3,5_-phenyl and C9-H_2,6_-phenyl), ,7.261-7.320 (m, 4H, C9-H2, 6-phenyl (2H) And *N-*Phenyl (2H)), 7.53-7.54 (m, 3H, *N-*phenyl); MS: m/z (%) 385 (M^+^, 50), 369 (5), 278 (100); IR (KBr): ν (cm^-1^) 3413, 2924, 1639, 1490

*9-(4-Methoxy-phenyl)-2, 3, 6, 7-tetrahydro-5H-cyclopenta [b] quinoline-1, 8-dione (7d). (C*_19_ *H*_17_ *NO*_3_ *)*

^1^H-NMR (CDCl_3_): δ 2.18-3.29 (m, 10H, Aliphatic), 3.86 (s, 3H, H-CH_3_), 6.96 (d, 2H, J = 6.5Hz, H_3,5_-phenyl), 7.09 (d, 2H, J = 6.5Hz , H_2,6_-phenyl); MS: m/z (%) 307 (M^+^, 100), 251 (5), 231 (2), 152 (2); IR (KBr): ν (cm^-1^) 3441, 2929, 1710, 1680

*9-(4-Bromophenyl)-2, 3, 6, 7-tetrahydro-4H-cyclopenta [b] quinoline-1, 8 (5H,9H)-dione (4e). (C*_18_ *H*_16_ *BrNO*_2_ *)*

^1^H-NMR (CD5l_3_): δ 1.95-2.65 (m, 10H, Aliphatic), 4.62 (s, 1H, H-C9), 7.09-7.11 (d, 2H, J = 8.5Hz ,C9-H_2,6_-phenyl), 7.35-7.37 (d, 2H, J = 8.5Hz , C9-H_3,5_-phenyl), 10.07 (s, 1H, H-NH), MS: m/z (%) 357/359 ((M^+^/M^+2^, 15/15), 309(12), 202(100), 198(20); IR (KBr): ν (cm^-1^) 3472, 2919, 1710, 1623

*9-(4-Bromo-phenyl)-4-phenyl-2, 3, 5, 6, 7, 9-hexahydro-4H-cyclopenta [b] quinoline-1, 8-dione (6e). (C*_24_ *H*_20_ *BrNO*_2_ *)*

^1^H-NMR (CDCl_3_): δ 2.12-2.50 (m, 10H, Aliphatic), 4.77 (s, 1H, H-C9), 7.26 (d, 2H, J = 8.5Hz , C9-H_2,6_-phenyl), 7.41 (d, 2H, J = 8.5Hz, C9-H_3,5_-phenyl), 7.51-7.59 (m, 4H, *N-*phenyl),

MS: m/z (%) 433/342 ((M^+^/M^+2^, 19/19), 278 (100), 248 (8), 192 (10); IR (KBr): ν (cm^-1^) 3416, 2919, 1644, 1488

*9-(4-Bromo-phenyl)-2, 3, 6, 7-tetrahydro-5H-cyclopenta [b] quinoline-1, 8-dione (7e). (C*_18_ *H*_14_ *BrNO*_2_ *)*

^1^H-NMR (CDCl_3_): δ 2.1-3.35 (m, 10H, Aliphatic), 7.07 (d, 2H, J = 8.5Hz , H_2,6_-phenyl), 7.52 (d, 2H, J = 8.5Hz , H_3,5_-phenyl); MS: m/z (%) 356/358 ((M^+^/M^+2^, 48/48), 354 (60), 165 (40), 69 (68), 55(100); IR (KBr): ν (cm^-1^) 3426, 2919, 1731, 1721, 1541

*9-(4-Nitrophenyl)-2, 3, 6, 7-tetrahydro-4H-cyclopenta [b] quinoline-1, 8 (5H,9H)-dione (4f). (C*_18_ *H*_16_ *N*_2_ *O*_4_ *)*

^1^H-NMR (CDCl_3_): δ 1.90-2.58 (m, 10H, Aliphatic), 4.77 (s, 1H, H-C9), 7.36-7.47 (m, 2H, C9-H_2,6_-phenyl), 7.99-8.10 (m, 2H, C9-H_3,5_-phenyl), 10.05 (s, 1H, H-NH), MS: m/z (%) 324 (M^+^, 58), 306 (25), 201 (100), 188 (58), IR (KBr): ν (cm^-1^) 3431, 3155, 1710, 1639

*9-(4-Nitro-phenyl)-4-phenyl-2, 3, 5, 6, 7, 9-hexahydro-4H-cyclopenta [b] quinoline-1, 8-dione (6f). (C*_24_ *H*_20_ *N*_2_ *O*_4_ *)*

^1^H-NMR (CDCl_3_): δ 2.12-2.52 (m, 10H, Aliphatic), 4.91 (s, 1H, H-C9), 7.56-7.58 (m, 4H, *N-*phenyl), 7.60-7.82 (m, 2H, C9-H_2,6_-phenyl), 8.11-8.23 (m, 2H, C9-H_3,5_-phenyl); MS: m/z (%) 400 (M^+^, 30), 278 (100), 193 (10), 76 (38); IR (KBr): ν (cm^-1^) 3426, 2914, 1721, 1644

*9-(4-nitrophenyl)-2, 3, 6, 7-tetrahydro-5H-cyclopenta [b] quinoline-1, 8-dione (7f) (C*_18_ *H*_14_ *N*_2_ *O*_4_ *)*

^1^H-NMR (CDCl_3_): δ 2.203-3.343 (m, 10H, Aliphatic), 7.252-7.270 (m, 2H, H_2,6_-phenyl), 8.272-8.318 (m, 2H, H_3,5_-phenyl); MS: m/z (%) 322 (M^+^, 100), 294 (58), 248 (46), 220 (30); IR (KBr): ν (cm^-1^) 3426, 2919, 1731, 1700, 1552, 1501

*9-(2-Nitrophenyl)-2, 3, 6, 7-tetrahydro-4H-cyclopenta [b] quinoline-1, 8 (5H,9H)-dione (4g). (C*_18_ *H*_16_ *N*_2_ *O*_4_ *)*

^1^H-NMR (CDCl_3_): δ 1.90-2.54 (m, 10H, Aliphatic), 5.5 (s, 1H, H-C9), 7.3-7.8 (m, 4H, C9-phenyl), 10.0 (s, 1H, H-NH), MS: m/z (%) 324 (M^+^, 5), 307 (20), 202 (100), 188 (20), IR (KBr): ν (cm^-1^) 3446, 3262, 2950, 1680, 1644

*9-(2-Nitro-phenyl)-4-phenyl-2, 3, 5, 6, 7, 9-hexahydro-4H-cyclopenta [b] quinoline-1, 8-dione (6g). (C*_24_ *H*_20_ *N*_2_ *O*_4_ *)*

^1^H-NMR (CDCl_3_): δ 1.82-2.49 (m, 10H, Aliphatic), 5.58 (s, 1H, H-C9), 7.38-7.79 (m, 9H, Aromatic); MS: m/z (%) 400 (M^+^, 18), 383 (78), 323 (30), 277 (100); IR (KBr): ν (cm^-1^) 2914, 1685, 1639

*9-(3-Bromophenyl)-2, 3, 6, 7-tetrahydro-4H-cyclopenta [b] quinoline-1, 8 (5H, 9H)-dione (4h). (C*_18_ *H*_16_ *BrNO*_2_ *)*

^1^H-NMR (CDCl_3_): δ 1.95-2.65 (m, 10H, Aliphatic), 4.63 (s, 1H, H-C9), 7.12-7.3 (m, 4H, C9-phenyl), 10.10(s, 1H, H-NH), MS: m/z (%) 357/359 ((M^+^/M^+2^, 10/10), 308 (35), 199 (100), 78 (20); IR (KBr): ν (cm^-1^) 3446, 3252, 2919, 1741 1639

*9-(3-Bromo-phenyl)-4-phenyl-2, 3, 5, 6, 7, 9-hexahydro-4H-cyclopenta [b] quinoline-1, 8-dione (6h). (C*_24_ *H*_20_ *BrNO*_2_ *)*

^1^H-NMR (CDCl_3_): δ 1.84-2.50 (m, 10H, Aliphatic), 4.81 (s, 1H, H-C9), 7.21-7.47 (m, 4H, *N-*phenyl), 7.49-7.59 (m, 5H, C9-phenyl)

MS: m/z (%) 433/435 ((M^+^/M^+2^, 50/50), 277 (100), 193 (10), 77 (22); IR (KBr): ν (cm^-1^) 3421, 2924, 1644, 1567

*Cytotoxicity section*

*Reagents and chemicals*

RPMI 1640, fetal bovine serum (FBS), trypsin and phosphate buffered saline (PBS) were purchased from Biosera (Ringmer, UK). The 3-(4, 5-Dimethylthiazol-2-yl)-2, 5-diphenyltetrazolium bromide (MTT) was obtained from Sigma (Saint Louis, MO, USA) and penicillin/streptomycin was purchased from Invitrogen (San Diego, CA, USA). Doxorubicin and dimethyl sulphoxide were obtained from EBEWE Pharma (Unterach, Austria) and Merck (Darmstadt, Germany), respectively.

*Cell lines and maintenance of human cell lines*

HeLa (human cervical adenocarcinoma), LS180 (human colon adenocarcinoma), MCF-7 (human breast adenocarcinoma) and Raji (human B lymphoma) cells were obtained from the National Cell Bank of Iran (Pasteur Institute, Tehran, Iran). All cell lines were maintained in RPMI 1640 supplemented with 10% FBS, and 100 units/mL penicillin-G and 100 µg/mL streptomycin. Cells were grown in monolayer cultures, except for Raji cells, which were grown in suspension, at 37°C in humidified air containing 5% CO_2_.

*MTT-based cytotoxicity assay*

Cell viability following exposure to synthetic compounds was estimated by using the MTT reduction assay (21-23). MCF-7 and Raji cells were plated in 96-well microplates at a density of 5 × 10^4^ Cells/mL (100 μL per well). LS180 and HeLa cells were plated at densities of 1 × 10^5 ^and 2.5 × 10^4^ Cells/mL, respectively. Control wells contained no drugs and blank wells contained only growth medium for background correction. After overnight incubation at 37°C, half of the growth medium was removed and 50 μL of medium supplemented with 4 different concentrations of synthetic compounds in the range of 1-100 µ M (1-50 µM for compounds 7a, 4b, 7b, 6d, 4f and 4g) were added in duplicate. Plates with Raji cells were centrifuged before this procedure. Compounds were all first dissolved in DMSO and then diluted in medium so that the maximum concentration of DMSO in the wells was 0.5%. Cells were further incubated for 72 h, except for HeLa cells, which were incubated for 96 h. At the end of the incubation time, the medium was removed and MTT was added to each well at a final concentration of 0.5 mg/mL and plates were incubated for another 4 h at 37°C. Then, formazan crystals were solubilized in 200 μL DMSO. The optical density was measured at 570 nm with background correction at 655 nm using a Bio-Rad microplate reader (Model 680). The percentage of inhibition of viability compared to control wells was calculated for each concentration of the compound and IC_15_ and IC_30_ values (24) were calculated with the CurveExpert software version 1.34 (for Windows). Each experiment was repeated 4 times. Data are presented as mean ± SD. 

## Results and Discussion

*Chemistry (synthesis of compounds)*

**Figure 1:**
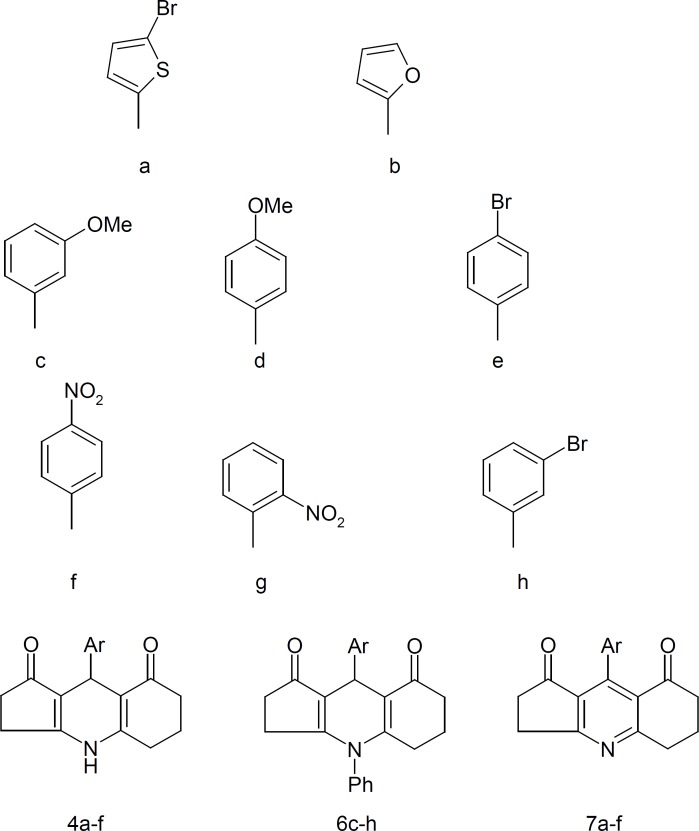
Chemical structures of cyclopenta [*b*] quinoline-1, 8-dione derivatives

In this project, 20 analogues of cyclopenta[b]quinoline-1, 8-dione were synthesized (Figure 1). 

The structures suggested for 4a-h, 6c-h and 7a–f have been confirmed by spectroscopic data using NMR, FT-IR and MS instruments. IR spectra were recorded on a Nicolet FT-IR Magna 550 spectrophotometer. ^1^H NMR spectra were measured using a Bruker FT-80 or FT-500 MHz, and chemical shifts were expressed as δ- values (ppm) against tetramethylsilane as internal standard. The mass spectra were run on a Finnigan TSQ-70 spectrometer at 70 eV.

*Cytotoxicity*

The cytotoxic activities of newly synthesized derivatives were assessed in 4 human cancer cell lines and IC_15_ and IC_30 _values were calculated for each derivative (Table 1).

**Table 1:** Cytotoxic activity of newly synthesized compounds assessed by the MTT reduction assay.

**Compound**	**HeLa cells**		**LS180 cells**		**MCF-7 cells**		**Raji cells**	
	**IC**_15 _ **(μM)**	**IC**_30_ **(μM)**	**IC**_15 _ **(μM)**	**IC**_30_ **(μM)**	**IC**_15 _ **(μM)**	**IC**_30 _ **(μM)**	**IC**_15 _ **(μM)**	**IC**_30_ **(μM)**
4a	17.6 ± 8.7	> 100	> 100	> 100	17.1 ± 8.9	> 100	71.2 ± 9.8	> 100
**7a**	39.0 ± 7.3	> 50	> 50	> 50	6.1 ± 4.8	> 50	> 50	> 50
**4b**	> 50	> 50	> 50	> 50	8.4 ± 6.8	27.6 ± 18.1	2.0 ± 0.2	6.7 ± 1.9
**7b**	17.9 ± 8.6	41.3 ± 23.1	> 50	> 50	20.3 ± 1.5	> 100	2.2 ± 0.6	6.6 ± 5.2
**4c**	18.0 ± 17.5	> 100	> 100	> 100	36.0 ± 44.2	> 100	8.9 ± 4.2	> 100
**6c**	42.8 ± 15.6	> 100	> 100	> 100	13.3 ± 21.7	> 100	4.2 ± 1.9	41.5 ± 32.8
**7c**	> 100	> 100	26.2 ± 0.6	> 100	11.1 ± 5.3	> 100	3.2 ± 1.3	10.0 ± 6.6
**4d**	17.4 ± 13.6	82.9 ± 71.7	> 100	> 100	8.1 ± 6.2	29.9 ± 15.5	> 100	> 100
**6d**	12.3 ± 2.5	> 50	> 50	> 50	> 50	> 50	6.0 ± 2.0	12.3 ± 2.0
**7d**	74.3 ± 5.0	> 100	> 100	> 100	27.5 ± 21.3	> 100	7.7 ± 8.2	63.0 ± 80.9
**4e**	4.4 ± 1.8	27.8 ± 14.7	> 100	> 100	39.0 ± 5.6	69.7 ± 13.0	15.2 ± 20.6	36.1 ± 35.9
**6e**	20.0 ± 2.5	47.4 ± 18.5	> 100	> 100	12.2 ± 17.3	28.6 ± 23.4	> 100	> 100
**7e**	> 100	> 100	> 100	> 100	17.6 ± 19.7	> 100	12.2 ± 18.1	34.3 ± 50.6
**4f**	12.7 ± 7.6	35.8 ± 21.3	> 50	> 50	5.7 ± 3.2	> 50	21.0 ± 24.5	> 50
**6f**	43.2 ± 14.4	>100	>100	>100	8.4 ± 6.1	25.9 ± 25.9	14.6 ± 15.6	>100
**7f**	36.7 ± 31.9	51.1 ± 33.6	>100	>100	72.5 ± 28.5	>100	>100	>100
**4g**	8.5 ± 5.5	19.1 ± 10.2	32.5 ± 15.4	> 50	14.9 ± 14.1	36.9 ± 32.1	4.3 ± 2.9	36.8 ± 32.3
**6g**	11.6 ± 9.4	> 100	> 100	> 100	20.4 ± 13.7	71.1 ± 46.3	14.0 ± 4.6	> 100
**4h**	21.1 ± 0.9	88.5 ± 33.7	> 100	> 100	28.7 ± 17.8	> 100	19.6 ± 13.1	47.4 ± 21.3
**6h**	13.4 ± 5.6	24.4 ± 6.8	31.9 ± 18.5	62.2 ± 22.1	24.9 ± 20.2	45.9 ± 40.8	2.4 ± 0.6	8.2 ± 4.5
**Doxorubicin**	0.027 ± 0.019	0.052 ± 0.032	0.015 ± 0.005	0.034 ± 0.009	0.009 ± 0.005	0.027 ± 0.011	0.048 ± 0.064	0.079 ± 0.103

Values represent the mean ± SD of 3-4 different experiments. Compounds were tested at the maximum final concentration of 100 µ M, except for compounds 7a, 4b, 7b, 6d, 4f and 4g, which were tested at 50 µ M due to lower. solubility

 On the basis of IC_15_ and IC_30_ values, it is obvious that most of compounds have weak effect on the different cell lines, since in most cases, the IC_50_ of these analogues were higher than 50 or 100 µ M.

The most and least potent compounds in each cell line were identified. In HeLa cell line, only two compounds, 6 h and 7f, had an IC_50_ lower than 100 µ M which their IC_50_ values were 55.4 and 84.0, respectively. Therefore, the most potent compounds in this cell line based on IC_50_ and IC_30_ in a decreasing order of efficiency were 6h > 7f > 4g > 7e. The weakest compounds were 7c and 7e which had IC_15 _and IC_30_ values higher than 100 μM.

In LS180 cell line, It is completely clear that the effect is negligible since only three compounds, 7c, 4g and 6h**, **had the IC_15_ lower than 50 or 100 μM (26.2, 32.5, and 31.9, correspondingly) and only one of them, 6h, showed an IC_30_ of 62.2 μM. 

In MCF-7 cells, the most potent compounds were 6f, 4b, 6b and 4d which their IC_30_ values were in the range of 25-30 μM. Unfortunately, in this cell line, none of compounds possessed IC_50 _of lower than 100 or 50 μM. Compound 6d, whose IC_15_ and IC_30_ values were both greater than 50 µ M was the weakest compound, although, the low solubility of compound prevented from a clear conclusion. It seems that these compounds had the highest effect on Raji cell line, as three compounds, 4b, 6d, and 6h, had IC_50_ of lower than 100 μM (25.8, 28.9, and 33.1, correspondingly). On the other hand, Compounds 7f, 6e, 4d, and 7a had IC_15 _and IC_30 _values of higher than 50 or 100 μM.

For the point of cell lines, it seems that these derivatives had a very weak effect on LS-180 cell line, since none of compounds did not show IC_30 _and IC_50 _values of lower than 50 or 100 μM (except one compound, 6h). Effects on the other three cell lines were to some extent similar. Therefore, it seems that the introduction of phenyl moiety on nitrogen of central quinoline ring might improve cytotoxic activity of compounds in most cases. 

## Conclusion

In the present study, a set of cyclopenta [b] quinoline-1, 8-dione derivatives were designed and synthesized and their cytotoxic activity of these compounds was evaluated *in-vitro *on four different cell lines including HeLa, LS180, MCF-7 and Raji. Although the results of cytotoxic activity evaluation demonstrated that the *in-vitro *anti cancer effect of synthesized compounds are mainly low, it seems that this structure can be used as a novel cytotoxic scaffold for further modification and design of novel potent compounds. 

**Table 2:** IC_50_ of Doxorubicin on different cell line

**IC**_50_ **(μM) of Doxorubicin**	**Cell line**	**References**
0.099	LS-180	24
0.056	MCF-7	25
0.31	Hela	26
